# Computational Study of Interdependence Between Hemagglutinin and Neuraminidase of Pandemic 2009 H1N1

**DOI:** 10.1109/TNB.2015.2406992

**Published:** 2015-03-02

**Authors:** Wei Hu

**Affiliations:** Houghton CollegeDepartment of Computer Science NY USA 14744

**Keywords:** Hemagglutinin, influenza, mutation, neuraminidase, pandemic 2009 H1N1, receptor binding specificity

## Abstract

Influenza type A viruses are classified into subtypes based on their two surface proteins, hemagglutinin (HA) and neuraminidase (NA). The HA protein facilitates the viral binding and entering a host cell and the NA protein helps the release of viral progeny from the infected cell. The complementary roles of HA and NA entail their collaboration, which has important implications for viral replication and fitness. The HA protein from early strains of pandemic 2009 H1N1 of swine origin preferentially binds to human type receptors with a weak binding to avian type receptors. This virus caused several human deaths in December 2013 in Texas, USA, which motivated us to investigate the changes of genetic features that might contribute to the surged virulence of the virus. Our time series analysis on the strains of this virus collected from 2009 to 2013 implied that the HA binding preference of this virus in USA, Europe, and Asia has been the characteristic of swine H1N1 virus since 2009. However, its characteristic of seasonal human H1N1 and its binding avidity for avian type receptors both were on steady rise and had a clear increase in 2013 with American strains having the sharpest surge. The first change could enhance the viral transmission and replication in humans and the second could increase its ability to cause infection deep in lungs, which might account for the recent human deaths in Texas. In light of HA and NA coadaptation and evolutionary interactions, we also explored the NA activity of this virus to reveal the functional balance between HA and NA during the course of virus evolution. Finally we identified amino acid substitutions in HA and NA of the virus that were critical for the observed evolution.

## I. Introduction

Influenza viruses can infect humans, swine, and avian species. Influenza A virus has an envelope, which is subtyped according to its two surface proteins, haemagglutinin (HA) and neuraminidase (NA). Both proteins recognize the glycan receptors on host cells, and they help the viral entry into and release of virions from host cells respectively. Wild aquatic birds are the reservoir of all influenza viruses found in different species [1]. HA is further cleaved into protein HA1 and protein HA2, the first of which binds to cellular receptors and the second mediates membrane fusion [2]. The HA and NA proteins play important roles in virulence, host specificity, and the human immune response. A functional balance of HA binding and NA cleavage is observed in the life cycle of human viruses, which is also needed for a zoonotic virus to cross the species boundary to efficiently transmit and reproduce in humans [3], [4].

Seasonal influenza viruses tend to bind receptors found on cells in the nose, throat, and upper airway to infect the respiratory tract of humans. The receptor binding site of HA is composed of three major structural elements: a 190-helix, a 220-loop, and a 130-loop (H3 numbering) [5]. The HAs of various viruses have different specificities for recognizing the sialic acids (SA) linked to galactose on the surface of host cells. The HAs of human viruses preferentially bind to oligosaccharides that terminate with sialic acid linked to galactose by $\alpha 2,6$-linkages (human type receptors), while the HAs of avian influenza favor oligosaccharides that terminate with a sialic acid linked to galactose by $\alpha 2,3$-linkages (avian type receptors). In aquatic birds avian type receptors dominate in tracheal epithelial cells, but in terrestrial birds both avian-type and human-type receptors are detected in their tracheal epithelial cells [6]. Also pigs have both human and avian receptors [7]. The $\alpha 2,6$ receptors are extensively detected in all areas of the respiratory tract of pigs with an average of 80–100% at the epithelial cells. On the contrary, the $\alpha 2,3$ receptors are not present at epithelial cells of nose, trachea, and most bronchi, but are found in small amounts in bronchioles, and in alveoli reaching an average of 20–40% at the epithelial cells [8]. The $\alpha 2,6$ receptor cells are usually present in the upper airway of humans, while the $\alpha 2,3$ receptor cells are most in the lungs. These findings suggest swine and avian species that express both $\alpha 2,3$ and $\alpha 2,6$ receptors could serve as an intermediate host for the emergence of new viral strains to infect humans, since this kind of adaptation often requires successful interspecies transmission.

The pandemic 2009 H1N1 virus of swine origin emerged in Mexico in March 2009 and quickly became a worldwide health threat in 2009. It resulted from reassortment of several viruses of swine origin. In particular, its NA gene was derived from Eurasian avian-like swine H1N1, and the HA gene from a triple-reassortant virus circulating in North American swine [9], [10]. This virus caused over 284 000 deaths within the first year of the pandemic, although its infections were characterized with mostly mild symptoms [11]. Further, the epidemiology of this virus was unique as it had a bigger impact on younger adults and older children. Unlike seasonal human H1N1 viruses, which bind mainly to $\alpha 2,6$ receptors, the pandemic 2009 H1N1 virus could bind to both $\alpha 2,3$ and $\alpha 2,6$ receptors although the primary binding is $\alpha 2,6$ receptors [12], [13].

The functions of HA and NA requires coordination and interdependence of their activity. The HA from the early strains of human 2009 H1N1 demonstrated lower binding avidity than the swine progenitor HA, due to amino acid substitutions near the receptor binding site. At the same time, the virus acquired an NA enzyme of relatively low activity through reassortment. In contrast to this functional match, the swine progenitors often deviate from such a functional balance. Therefore, appropriately matched activities of HA and NA from zoonotic viruses might be indicators of transmission efficiency in humans [3], [4], [14], [15].

An existing balance between HA and NA, such as substrate specificity of NA and receptor binding specificity of HA, could be disturbed by various causes, such as reassortment, virus transmission to a new host, or therapeutic inhibition of neuraminidase, which could be restored by compensatory mutations in HA and NA [3], [16], [17]. In comparison of the two proteins, HA could bind to $\alpha 2,6$ or $\alpha 2,3$ receptors, whereas NA has a marked preference for $\alpha 2,3$ receptors although it can cleave both types [18]. However, the NA of the pandemic 2009 H1N1 shows a distinctive enzymatic profile, which hydrolyzes $\alpha 2,3$ receptors as efficiently as avian viruses and hydrolyzes $\alpha 2,6$ receptor as efficiently as classical swine viruses [19].

In the flu season of 2013, the predominant strain of influenza was pandemic 2009 H1N1 in the south-central United States, where several human deaths were reported in Texas (health.usnews.com and www.usatoday.com). In search of any altered molecular features from this virus that might lead to its recent increase of virulence as observed in Texas, we sought to discover, with a computational approach developed in [20]–[39], any variations in the HA binding patterns of the strains collected from 2009 to 2013. Due to their closely interacting roles, a balance needs to be maintained in the HA and NA functional activity, which could be mediated by genetic changes in HA and NA. Following this view, we also quantified the NA activity of this virus in association with the HA receptor binding.

## II. Matertials and Methdos

### A. Sequence Data

The HA and NA protein sequences of influenza viruses used in this study were retrieved from the EpiFlu Database (http://platform.gisaid.org) of the Global Initiative on Sharing Avian Influenza Data (GISAID) and the Flu Virus Database of NCBI (http://www.ncbi.nlm.nih.gov/genomes/FLU/Database). The beta information about these sequences, such as number of sequences, sampling period, geographic range, host, can be found in Table II in Section III. All the sequences were aligned with MAFFT [40]

**Table I:** The electron-ion interaction potential (eiip) of amino acids used to encode amino acids

Amino acid	EIIP		Amino acid	EIIP
L	0.0000		Y	0.0516
I	0.0000		W	0.0548
N	0.0036		Q	0.0761
G	0.0050		M	0.0823
E	0.0057		S	0.0829
V	0.0058		C	0.0829
P	0.0198		T	0.0941
H	0.0242		F	0.0946
K	0.0371		R	0.0959
A	0.0373		D	0.1263

### B. Informational Spectrum Method

The informational spectrum method (ISM) is a computational approach that can be employed to analyze protein sequences [41], [42]. The idea is to transform the protein sequences into numerical sequences based on electron-ion interaction potential (EIIP) of each amino acid (Table I).

The numerical sequence ${\rm x}({\rm m})$ of a protein sequence is transformed into the frequency domain using discrete Fourier transform (DFT). The DFT coefficients ${\rm X}({\rm n})$ are defined as

$$X\left(n \right) = \sum\limits ^{x}\left(m \right){e^{- j\left({2\pi /N} \right)nm}}\quad n = 1,2, \ldots {N \over 2}$$

where N is the length of sequence $x (m)$.

The energy density spectrum is defined as

$$S\left(n \right) = X\left(n \right){X^{*}}\left(n \right) = \vert X\left(n \right){\vert^{2}},\quad n = 1,2, \ldots.,{N \over 2}.$$

The informational spectrum (IS) of a sequence ${\rm x}({\rm m})$ comprises the frequencies and the amplitudes of its DFT. According to the ISM theory, the peak frequencies of IS of a protein sequence reflect its biological or biochemical functions. In this manuscript, we used F to represent the frequency of IS. The ISM was successfully applied to quantify the effects of HA mutations on the receptor binding preference in [25], [41], [42].

## III. Results

Our theme of this study was using ISM to elucidate any modifications of HA and NA proteins from pandemic 2009 H1N1 from 2009 to 2013. For this end, we also included the HA and NA of H1N1 from different species and regions, laying a foundation for our analysis of pandemic 2009 H1N1.

### A. IS of HA and NA of H1N1 From Different Species and Regions

To provide the background for the analysis based on ISM, we calculated the IS of each HA and NA of H1N1 from different species and regions, and took an average of their IS therein. The top two average IS frequencies, primary and secondary, of H1N1 HA and NA respectively from different species and regions were reported (Table II). In the previous work [41], [42], a product of their IS was employed to

**Table II:** Primary and secondary IS frequencies of HA and NA of h1n1 from different species and regions

Protein	Virus	First	Second	Number of sequences	Years of collection
HA	Pandemic 2009 Human H1N1 in USA	F(0.295)	F(0.055)	2670	2009-2013
NA	Pandemic 2009 Human H1N1 in USA	F(0.074)	F(0.346)	2670	2009-2013
HA	Pandemic 2009 Human H1N1 in Europe	F(0.295)	F(0.055)	1689	2009-2013
NA	Pandemic 2009 Human H1N1 in Europe	F(0.074)	F(0.346)	1689	2009-2013
HA	Pandemic 2009 Human H1N1 in Asia	F(0.295)	F(0.258)	1864	2009-2013
NA	Pandemic 2009 Human H1N1 in Asia	F(0.074)	F(0.346)	1864	2009-2013
HA	Pandemic 2009 Swine H1N1 in USA	F(0.295)	F(0.258)	116	2009-2013
NA	Pandemic 2009 Swine H1N1 in USA	F(0.074)	F(0.346)	116	2009-2013
HA	Pandemic 2009 Swine H1N1 in Europe	F(0.295)	F(0.258)	20	2009-2013
NA	Pandemic 2009 Swine H1N1 in Europe	F(0.074)	F(0.346)	17	2009-2011
HA	Pandemic 2009 Swine H1N1 in Asia	F(0.295)	F(0.258)	149	2009-2012
NA	Pandemic 2009 Swine H1N1 in Asia	F(0.074)	F(0.346)	116	2009-2012
HA	Seasonal Human H1N1 in USA	F(0.055)	F(0.236)	876	2000-2009
NA	Seasonal Human H1N1 in USA	F(0.373)	F(0.268)	876	2000-2009
HA	Swine H1N1 in USA	F(0.295)	F(0.055)	668	1930-2013
NA	Swine H1N1 in USA	F(0.373)	F(0.074)	668	1930-2013
HA	Avian H1N1 in USA	F(0.281)	F(0.295)	190	1980-2012
NA	Avian H1N1 in USA	F(0.168)	F(0.074)	190	1980-2012
HA	Seasonal Human H1N1 in Europe	F(0.055)	F(0.236)	230	2000-2009
NA	Seasonal Human H1N1 in Europe	F(0.373)	F(0.268)	250	2000-2009
HA	Swine H1N1 in Europe	F(0.295)	F(0.281)	116	1939-2012
NA	Swine H1N1 in Europe	F(0.074)	F(0.484)	169	1979-2013
HA	Avian H1N1 in Europe	F(0.295)	F(0.281)	28	1977-2009
NA	Avian H1N1 in Europe	F(0.074)	F(0.490)	35	1983-2009
HA	Seasonal Human H1N1 in Asia	F(0.055)	F(0.236)	1366	2000-2010
NA	Seasonal Human H1N1 in Asia	F(0.373)	F(0.268)	756	2001-2010
HA	Swine H1N1 in Asia	F(0.295)	F(0.281)	687	1974-2012
NA	Swine H1N1 in Asia	F(0.484)	F(0.074)	667	1977-2012
HA	Avian H1N1 in Asia	F(0.281)	F(0.295)	24	1976-2011
NA	Avian H1N1 in Asia	F(0.074)	F(0.240)	33	1977-2011

find the most prominent frequency, but here we sought to discover several top frequencies as Influenza viruses tend to display multiple binding patterns. The HA frequencies F(0.295) and F(0.055) were first analyzed in study of H1N1 [41], and F(0.236) and F(0.258) of HA were calculated in [42]. Although only the top two IS frequencies were selected in Table II, the actual top three HA frequencies of pandemic 2009 human H1N1 were F(0.295), F(0.055), and F(0.258) in USA, and F(0.295), F(0.258), and F(0.055) in Europe and Asia, while the swine H1N1 in USA had F(0.295) and F(0.055) as its top two frequencies, showing the HA binding of pandemic 2009 H1N1 was similar to that of North American swine H1N1 [9], [10]. Eurasian swine H1N1 HA binding patterns [F(0.295) and F(0.281)] were all avian like because F(0.281) was their secondary binding frequency. The avian like swine viruses emerged in Europe in the late 1970s after an avian virus was introduced to swine [19]. Seasonal human H1N1 in USA, Europe, and Asia all had the same top two HA IS frequencies F(0.055) and F(0.236) and the same NA frequencies F(0.373) and F(0.268).

The NA of pandemic 2009 H1N1 had its top three IS frequencies, F(0.074), F(0.347), and F(0.484). F(0.074) was the primary NA frequency of swine and avian H1N1 in Europe and avian H1N1 in Asia. F(0.484) was the primary of swine H1N1 in Asia and secondary of swine H1N1 in Europe. Our analysis here suggested that the NA sialidase activity of pandemic 2009 H1N1 was most similar to that of avian like Eurasian swine H1N1, which was in line with the NA origin of this virus [9], [10]. However, a study in [19] showed that the NA activity of this virus was closer to that of classical swine viruses than to that of avian, avian-like-swine, and seasonal human viruses.

### B. IS of HA and NA of H1N1 From Different Species in USA

**Fig. 1.**
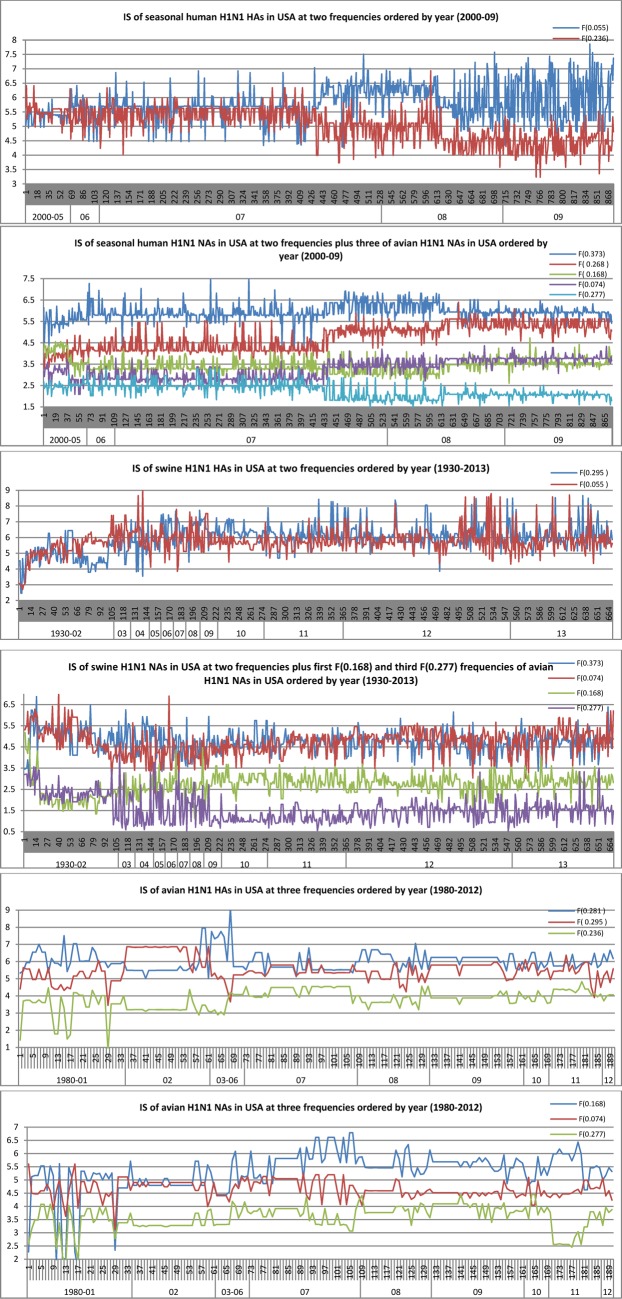
IS of HA and NA of h1n1 from different origins in usa, where the y-axis represents the amplitude of IS and the x-axis represents the sequence count and year.

Location is critical for spread and transmission of influenza. Here we conducted ISM on the HA and NA of H1N1 from various species in USA as the pandemic 2009 H1N1 emerged in North America first (Fig. 1). In addition to the IS information in section A, the IS analysis of the H1N1 viruses in USA in this section helped understanding the IS of HA and NA of pandemic 2009 H1N1 presented in the next section. The advantage of our work here was able to show the changing patterns of HA and NA and their correlation, if any, over a period of several years. We could see that the primary and secondary HA IS at F(0.055) and F(0.236) of seasonal H1N1 in USA remained at the same level (the top two ISs of NA kept a constant gap), but after 2007 HA IS at F(0.055) played a clear leading role (the leading role of NA IS at F(0.373) started to diminish). Also there was a fluctuation of the top two HA ISs from 2008 to 2009, but their gap remained a constant during these two years (the top two ISs of NA started to reduce their gap). In contrast, the top two ISs of HA and NA of swine H1N1 in USA kept mixing with no clear leader during the whole period of 1930–2013. A random pattern of HA and NA from avian H1N1 in USA could also be seen. One thing worth noting was the first and third NA ISs of avian H1N1 in USA were low in the NA activity of seasonal human and swine H1N1 in USA (Fig. 1),

which were quite different from the case of pandemic 2009 H1N1 in the next section.

### C. IS of HA and NA of Pandemic 2009 Human H1N1 in Europe and Asia

**Fig. 2.**
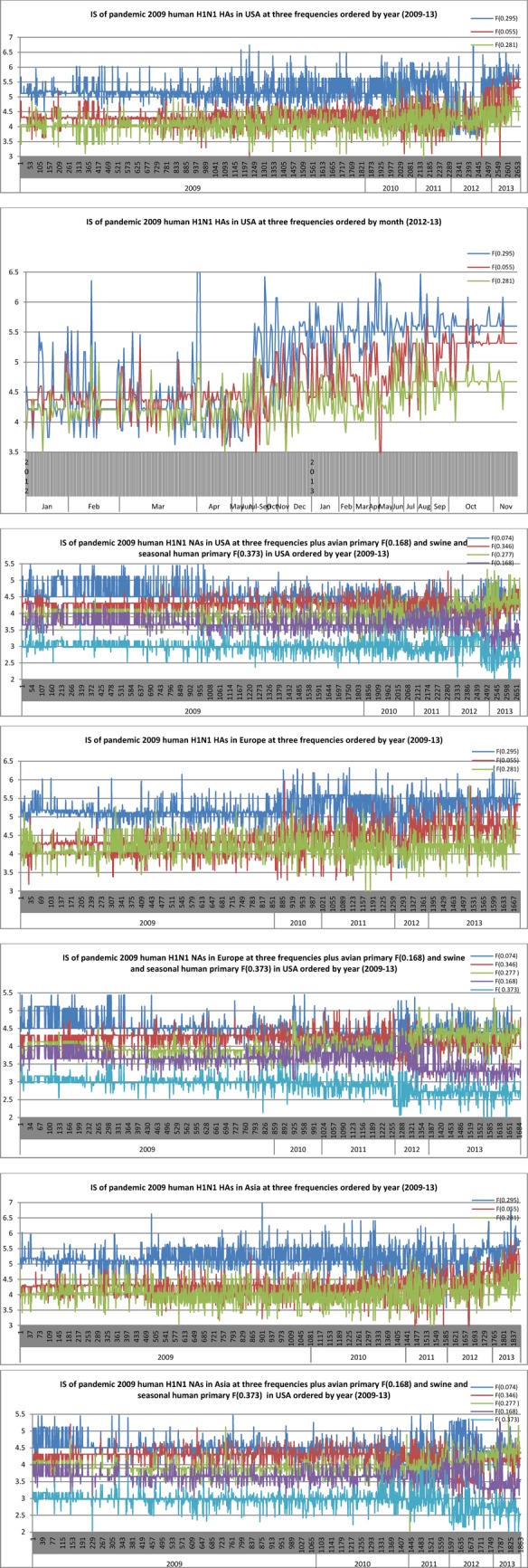
IS of HA and NA of pandemic 2009 h1n1 in europe and asia, where the y-axis represents the amplitude of IS and the x-axis represents the sequence count and year.

Our original aim was to find out any molecular changes of HA and NA of pandemic 2009 H1N1 in USA from 2009 to 2013 that might contribute to the recent human deaths caused by this virus in Texas. We thought it would be beneficial if we could include the strains of this virus from Europe and Asia as well to render a bigger picture. To find any changing patterns of HA binding specificity, some leading HA IS frequencies of pandemic 2009 human H1N1 in different regions were presented (Fig. 2). We did not plot the HA IS at F(0.258) in this section because it remained relatively stable from 2009 to 2013 compared to that at other top frequencies, even though it was one of the top frequencies of this virus. There were noticeable number of HA or NA sequences in 2009 that did not have the month and day information, so they were placed in the beginning of 2009 in our plot. Nonetheless, in any case, the HA and NA from the same virus isolate were placed at the same position of the plot to visualize any possible correlation between these two proteins over time.

The primary HA IS of this virus in USA and Asia arose in 2009, but this occurred in 2010 in Europe. As in the case of American seasonal H1N1, there seemed a trend, i.e., the primary HA IS went higher where the primary NA IS was lower. There was a clear drop of the HA ISs in 2012, with the drop in USA being the largest. These HA ISs then started to increase, which occurred for American HA ISs in July of 2012 (Fig. 2). Minus this drop, the primary HA IS kept stable. However, the HA ISs at F(0.055) and F(0.281) were on the steady rise from 2009 to 2013 with a sharp increase in 2013 for American strains. Accompanying this HA IS increase was rise of the NA ISs at F(0.346) and F(0.277) in USA, Europe, and Asia. F(0.281) was the primary HA IS frequency of American avian H1N1 (Fig. 1). F(0.277) was the third NA IS frequency of American avian H1N1 (Fig. 1) and F(0.346) was the second frequency and a characteristic frequency of pandemic 2009 H1N1 (Table II). It was interesting to see that the primary NA IS at F(0.166) of American avian H1N1 and that at F(0.373) of American swine and seasonal human H1N1 remained low in the NA activity of pandemic 2009 H1N1. Both NA ISs at F(0.346) and F(0.277) of pandemic 2009 H1N1 were in a quick decrease in 2013 while its NA ISs at F(0.346) and F(0.277) were on a rise.

In summary, the primary HA IS at F(0.295) (a swine H1N1 feature frequency) of this virus remained relatively stable throughout the period of 2009–2013, with one drop in 2012. Moreover, the HA ISs at F(0.055) (a seasonal human H1N1 feature frequency) and at F(0.281) (an avian H1N1 feature frequency) were on steady rise and had a clear increase in 2013 with the American strains having the sharpest surge (Fig. 2).

To go together with the variations of HA IS in time, the NA ISs of this virus at F(0.074), F(0.346), and F(0.277) were at three different levels in 2009. But they started to converge in 2010, and were well mixed in 2013. Remembered that F(0.277) was a top NA IS frequency of American avian H1N1 (Fig. 1). Another evident trend was that the NA ISs started a drop in 2012 at the primary frequency of avian H1N1 F(0.168) and at the primary frequency of seasonal human H1N1 F(0.373). The association of the changing patterns of HA and NA observed here called for an experimental approach to elucidate further their functional interdependence.

The primary NA IS of seasonal human H1N1 in USA had an average of 6.0, whereas that of pandemic 2009 H1N1 in USA, Europe, and Asia all had 4.5, which was close to 4.7 of the classical swine H1N1 in USA but still lower than 5.3 of avian H1N1 in USA (Figs. 1 and 2). This finding was matched by the experiments in [19]. Further, the NA ISs of avian H1N1 at F(0.168) and F(0.277) were low in swine H1N1 (Fig. 1), showing swine H1N1 hydrolyzed $\alpha 2,3$-linked sialoside less efficiently than did pandemic 2009 H1N1 [19].

### D. IS of HA and NA of Pandemic 2009 H1N1 in USA From An Early Strain in 2009 and A Recent Strain in 2013

**Fig. 3.**
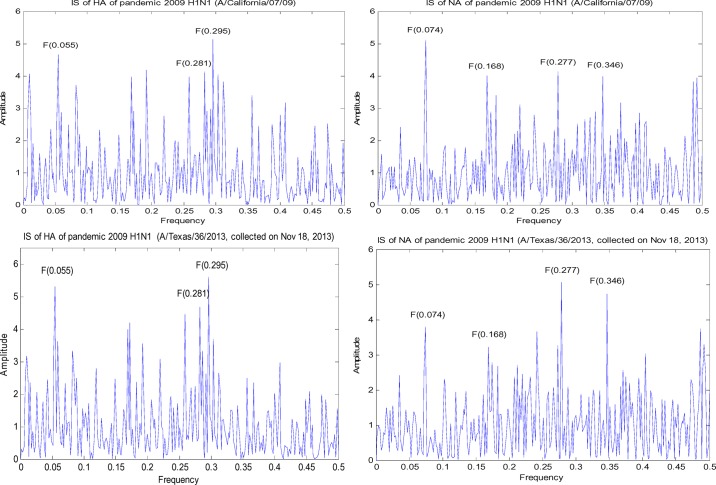
IS of HA and NA from two representative strains of pandemic 2009 h1n1 in usa, one at the start of 2009 and one at the end of 2013.

In Sections III-B and III-C, the stream of activity patterns of HA and NA from each virus was presented over a period of various years. In this section, we rendered a quick snapshot of the IS of HA and NA from pandemic 2009 H1N1 in USA at the two ends of this stream, one at the start of 2009 (A/California/7/2009) and one at the end of 2013 (A/Texas/36/2013), to show the whole IS in each case (Fig. 3). A/California/7/2009 was selected because it was one of the strains used in the composition for the Northern Hemisphere 2013–2014 influenza vaccine (http://www.cdc.gov/flu/about/season/vaccine-selection.htm), and A/Texas/36/2013 was chosen because it was collected on November 18, 2013, just before the human deaths caused by this virus in Texas in December, 3013. The alterations of their IS resulting from the slow variations in HA and NA were shown (Fig. 3). The HA IS of A/Texas/36/2013 at F(0.281) was increased to the third, and at the same time the NA ISs of A/Texas/36/2013 at F(0.277) and F(0.346) became the first and second respectively, compared to A/California/07/2009. The ISs of HA and NA from these two strains in Fig. 3 captured the status of the two ends well.

### E. Mutations in HA and NA of Pandemic 2009 H1N1 in USA that Caused the Change in Their Activity From 2009 To 2013

**Fig. 4.**
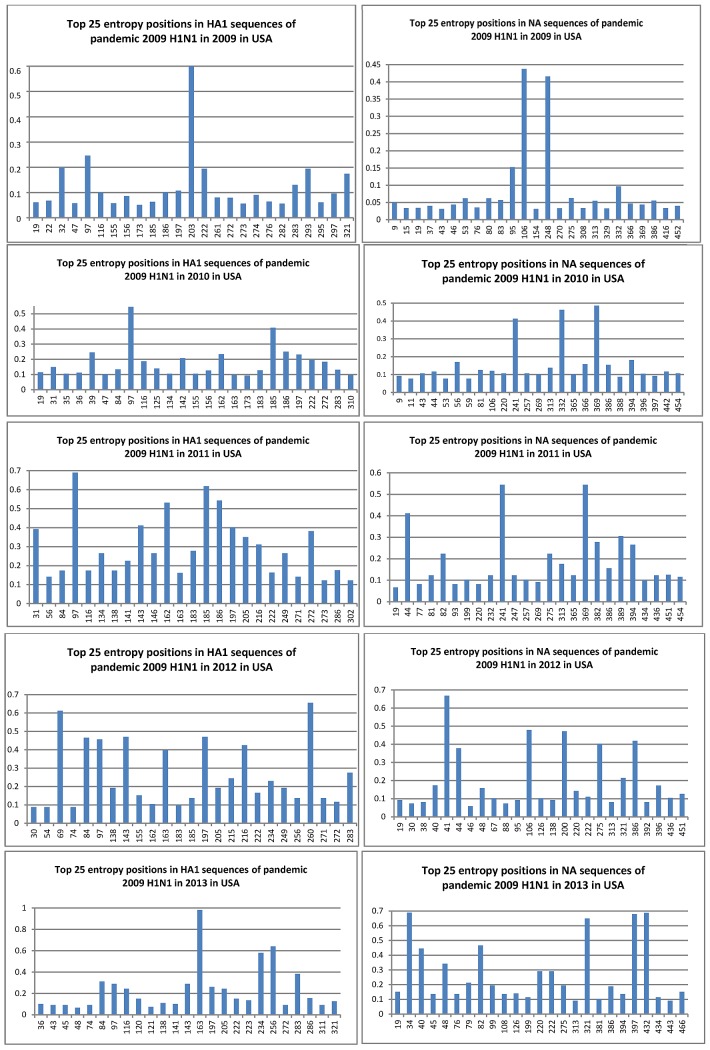
Entropy of HA and NA of pandemic 2009 h1n1 in USA by year from 2009 to 2013 showing the gradual changes of their amino acids.

Our first task here was to show the entropy [43] of HA and NA of pandemic 2009 H1N1 in each year from 2009 to 2013, as entropy could measure the propensity of amino acid change at a particular residue position (Fig. 4). The top 25 entropy positions of each year highlighted the slow shift of the genetic makeup of HA and NA of this virus. HA mutations S183P and S185T were found to increase the receptor binding avidity of HA, whereas A134T and A197T decreased that of HA [44]. Other mutations in HA that might increase the disease severity of this virus were reported in [45]. Our entropy analysis detailed the potential change of the amino acids at these positions from year to year, say, the entropy of HA at position 163 (Fig. 4).

Our second task was to identify the amino acid substitutions in HA and NA that could actually cause the gradual change of their activity patterns from 2009 to 2013. We reported respectively the top 5 positions in HA and NA recognized by Random Forest [46], [47] to distinguish the sequences in 2009 from those in 2013.

**Table III:** Important amino acid substitutions found by random forest in HA and NA that led to the shift of HA and NA activity of pandemic 2009 h1n1 from 2009 to 2013

Position in HA	97	163	185	256	283	Position in NA	44	106	200	241	369
2009	D	K	S	A	K	2009	N	I	N	V	N
2013	N	*Q*	T	T	E	2013	S	V	S	I	K

The input to Random Forest was protein sequences and the use of Random Forest was for feature ranking, not for classification. The actual amino acids at each of these positions in 2009 and 2013 were shown (Table III) and the impact on the IS of HA and NA at leading frequencies by these amino acid substitutions was also presented (Table IV), which assessed the validity of our finding. The calculation in Table IV detailed the individual as well as collective contribution to the IS alterations by these mutations. It was noted that A/California/7/2009 had 106 V in its NA already.

**Fig. 5.**
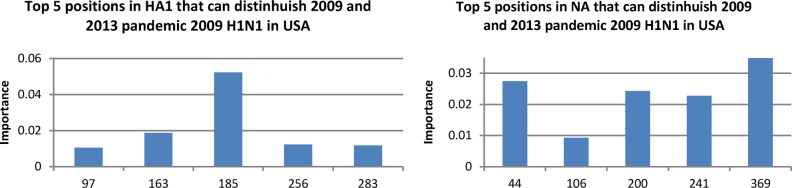
Top positions measured by random forest in HA and NA of pandemic 2009 h1n1 in USA that could differentiate the sequences in 2009 from those in 2013.

**Table IV:** Impact on the IS at leading frequencies of HA and NA of pandemic 2009 h1n1 in USA by the critical amino acid substitutions in HA and NA found by random forest (the baseline was (a/california/7/2009)

	HA frequency					NA frequency			
Mutation in HA	F(0.295)	F(0.055)	F(0.258)	F(0.281)	Mutation in NA	F(0.074)	F(0.277)	F(0.168)	F(0.346)
Baseline	5.1361	4.6632	3.9709	4.1307	Baseline	5.1212	4.1260	4.0124	4.0083
D97N	5.6745	4.9285	4.3360	4.1786	N44S	4.7653	4.0854	3.7631	3.8580
K163Q	5.2250	4.8502	3.8933	4.2044	N200S	5.5389	4.5002	3.7062	4.0116
S185T	5.1037	4.7084	3.9216	4.1865	V241I	5.0897	4.1413	4.0358	4.0295
A256T	5.1326	4.9855	3.8067	4.4266	N369K	4.9687	4.2380	4.1714	3.9493
K283E	5.0680	4.8345	4.0789	4.2870	N44S, N200S, V241I, N369K	4.9836	4.5973	3.6310	3.8265
D97N, K163Q, S185T, A256T, K283E	5.6422	5.7017	4.1145	4.7916	Total increase compared to baseline	−0.1376(2.7%)	0.4713 (10.3%)	−0.3814 (9.5%)	−0.1818(4.5%)
Total increase compared to baseline	0.5061(9%)	1.0385(18%)	0.1436(0.04%)	0.6609(14%)					

HA mutations D222G or D222E in the receptor binding site of pandemic 2009 H1N1 HA were found sporadically, and D222G was correlated with cases of severe or fatal disease and its changed the HA binding preference [48]. When D222G was applied to an early strain of pandemic 2009 H1N1, A/California/04/09, it showed a modest reduction in the binding avidity to $\alpha 2,6$ receptors and an increase in the binding to $\alpha 2,3$ receptors in comparison with wild-type virus [49]. Therefore, D222G acquired dual receptor specificity for $\alpha 2,3$ and $\alpha 2,6$ receptors, which implied more efficiency in human transmission and enhanced ability to infect human lungs and might help explain why some patients that contracted it also developed more serious lung infections.

**Table V:** Impact on the average HA is at four leading frequencies of pandemic 2009 h1n1 sequences in USA by mutation d22g

Receptor binding type associated with the leading frequencies	Human Type	Human Type	Human Type	Avian Type
Four leading frequencies	F(0.295)	F(0.055)	F(0.258)	F(0.281)
HAs containing 222D (n-2903)	5.1800	4.3514	4.3432	4.0843
HAs containing 222G (n=41)	4.9417	3.9848	3.807	4.7867
After applying D222G to HAs containing 222D (n=2903)	5.0809	3.9399	3.845	4.7092

We applied D222G to a collection of HA sequences from pandemic 2009 H1N1 in USA, of which 2903 contained 222D and 41 had 222G (Table V). Our findings on this mutation D222G (Table V) demonstrated its effect to decrease human binding and increase avian binding, matching the experimental results in [49] perfectly. Similar experimental results on other strains of this virus were reported in [50].

## IV. Conclusion

The recent human deaths in Texas caused by pandemic 2009 H1N1 in December 2013 alerted us to take a closer look at this virus again. We wondered what change has taken place in this virus from 2009 to 2013 that might contribute to the observed increased virulence. Our initial plan was to examine the HA and NA sequences of pandemic 2009 H1N1 in USA to find the causes for the Texas cases. But soon we realized that we needed to include more sequences from different species and regions to really offer a bigger picture to better understand this virus. With sequences of H1N1 from different species and various regions, our study allowed for new insight into the evolution of this pandemic virus in great detail.

We first analyzed the HA and NA sequences from seasonal human, swine, avian viruses in USA, Europe, and Asia collected from a period of various years. Then we evaluated the HA and NA sequences of pandemic 2009 H1N1 collected from 2009 to 2013 in USA, Europe, and Asia. Our time series analysis showed that the HA binding preference of this virus has been the characteristic of swine H1N1 virus since 2009; however, its characteristic of seasonal human H1N1 and its binding to avian type receptors both were on steady rise and had an increase in 2013 with American strains having the sharpest surge. The first change could improve the viral transmission and replication in humans and the second could enhance its ability to cause infection deep in lungs, which might contribute to the increased virulence of this virus in Texas. In light of the closely interacting roles of HA and NA, we further studied the NA activity of this virus to reveal the interdependence between HA and NA during the virus evolution from 2009 to 2013. Some of our findings on HA and NA were supported by experimental results. We also identified amino acid substitutions in HA and NA of the virus that were critical for the observed evolution.

## Supplementary Material

Color versions of one or more of the figures in this paper are available online at http://ieeexplore.ieee.org.
